# Romiplostim in children with newly diagnosed or persistent primary immune thrombocytopenia

**DOI:** 10.1007/s00277-021-04590-0

**Published:** 2021-07-26

**Authors:** John D. Grainger, Thomas Kühne, Jane Hippenmeyer, Nichola Cooper

**Affiliations:** 1 Department of Haematology, University of Manchester, Royal Manchester Children’s Hospital, Manchester, UK; 2 Oncology/Hematology, University Children’s Hospital Basel, Basel, Switzerland; 3 Amgen, Rotkreuz, Switzerland; 4 Centre for Haematology, Department of Immunology and Inflammation, Imperial College London, London, UK

**Keywords:** Thrombopoietin receptor agonist, Bleeding, Corticosteroids, Romiplostim, Eltrombopag, Rituximab

## Abstract

Immune thrombocytopenia (ITP) is a disease of heterogenous origin characterized by low platelet counts and an increased bleeding tendency. Three disease phases have been described: newly diagnosed (≤ 3 months after diagnosis), persistent (> 3–12 months after diagnosis), and chronic (> 12 months after diagnosis). The majority of children with ITP have short-lived disease and will not need treatment. For children with newly diagnosed ITP, who have increased bleeding symptoms, short courses of steroids are recommended. In children who do not respond to first-line treatment or who become steroid dependent, thrombopoietin receptor agonists (TPO-RAs) are recommended because of their efficacy and safety profiles. In this narrative review, we evaluate the available evidence on the use of the TPO-RA romiplostim to treat children with newly diagnosed or persistent ITP and identify data from five clinical trials, five real-world studies, and a case report. While the data are more limited for children with newly diagnosed ITP than for persistent ITP, the collective body of evidence suggests that romiplostim is efficacious in increasing platelet counts in children with newly diagnosed or persistent ITP and may result in long-lasting treatment-free responses in some patients. Furthermore, romiplostim was found to be well tolerated in the identified studies. Collectively, the data suggest that earlier treatment with romiplostim may help children to avoid the side effects associated with corticosteroid use and reduce the need for subsequent treatment.

## Introduction

Primary immune thrombocytopenia (ITP) is an autoimmune disease characterized by a transient or persistent decrease in platelet counts (< 100 × 10^9^/l) [1, 2]. ITP is a rare condition affecting individuals of all ages with an estimated yearly incidence of approximately 1.9–6.4 per 100,000 in children [3, 4]. Pediatric ITP is more likely to be short-lived and resolves spontaneously than adult disease [5]. However, there is a subset of children for whom this is not the case, and it is clear that a high level of heterogeneity exists within the pediatric population, in terms of clinical presentation, treatment responses, and remission rates [5].

The most common symptom of ITP is increased bleeding tendency, which frequently presents as bruising and petechiae [6, 7]. Severe mucosal bleeding episodes can occur in some children; these are preferably managed in hospital [8]. Severe bleeding has been reported in up to 20% of cases (depending on the definition of severe bleeding) [9, 10], while life-threatening intracranial hemorrhage is very rare, occurring in < 1% of cases [9, 11, 12]. ITP can have a profound negative impact on the health-related quality of life (HRQoL) of children [13–15].

Historically, an arbitrary distinction with no biological basis has been made between “acute” and “chronic” ITP, with the latter typically defined as a disease duration of ≥ 6 months [6]. In 2009, an international working group proposed that ITP should instead be considered to have three phases, defined as newly diagnosed (≤ 3 months after diagnosis), persistent (> 3–12 months after diagnosis), and chronic (> 12 months after diagnosis) [1]. These definitions were created because of the reduced likelihood of spontaneous remission with increased duration of ITP (highlighting to clinicians that irreversible treatments such as splenectomy should be avoided for patients who could still achieve remission) [5, 6, 16]. The change in the definition has posed many challenges in clinical practice, such as how to best treat children within the new framework when original drug indications and clinical trial data were based on the old definition.

Various management options have been used for children with newly diagnosed or persistent ITP. In children requiring intervention, the standard first-line therapy has generally been corticosteroids with the addition of intravenous immunoglobulin (IVIg) or anti-D to manage acute bleeding episodes; however, long-term corticosteroid use is associated with a number of serious adverse events, and so its duration of use should be limited [6, 7, 17]. A range of subsequent therapies are in clinical use, with thrombopoietin receptor agonists (TPO-RAs), which stimulate platelet production via activation of the c-Mpl receptor [18], being key second-line agents recommended by recent international and national guidelines [6, 7, 17].

Romiplostim is a TPO-RA that is approved in Europe for the treatment of chronic ITP in children ≥ 1 year of age who are refractory to other treatments (e.g., corticosteroids and immunoglobulins) [19]. Notably, the European label of romiplostim in adult patients has recently been updated to remove the chronic disease restriction; romiplostim is approved for all adults with primary ITP who are refractory to other treatments [19]. In the USA, romiplostim is approved for children ≥ 1 year of age with ITP for ≥ 6 months who have had insufficient response to corticosteroids, immunoglobulins, or splenectomy [20]. Numerous studies have demonstrated the efficacy of romiplostim in increasing platelet counts, together with its low toxicity and high tolerability in children with chronic ITP [21–28]. Additionally, romiplostim may induce sustained treatment-free responses in a subset of children with chronic ITP [25, 27].

The labels of romiplostim in Europe and the USA were granted based on clinical trials that were mainly performed using the historical definition of chronic ITP (≥ 6 months after diagnosis). However, under the current definitions for ITP phases [1], some of the children in the pivotal trials of romiplostim who had ITP for ≥ 6 months but < 1 year and were previously classed as having chronic ITP would now be classified as having persistent ITP. Ideally, the labels should reflect the populations of the trials on which the approvals were based. Furthermore, while romiplostim is approved for treating children with chronic ITP in Europe, a growing number of real-world studies suggest that romiplostim is being used in clinical practice to treat newly diagnosed or persistent ITP in children who do not respond to first-line therapies. The aim of this narrative review is to collate and evaluate all the available evidence from randomized clinical trials, real-world studies, and case reports on the use of romiplostim for treating children with newly diagnosed or persistent ITP.

## Romiplostim for children with newly diagnosed ITP

### Guidelines

Major regional and international guidelines have been published over the past few years that provide updated guidance on the treatment of children with newly diagnosed ITP [6, 7, 17]. In 2019, an updated International Consensus Report was published by an expert panel of 22 members around the world [7]. The report included a critical review of manuscripts up until July 2018, together with consensus-based recommendations for adults and children. Also in 2019, the American Society of Hematology (ASH) published an update of their 2011 guidelines for ITP [17]. The guidelines were developed by a multidisciplinary panel of 17 members, who assessed evidence up until May 2017 and agreed on 21 graded consensus recommendations covering management of ITP in adults and children. Finally, a joint working group of European hematology societies in Germany, Austria, and Switzerland updated their guidelines in 2018, based on all relevant manuscripts on ITP until November 2017 [6].

At diagnosis, children and adolescents with ITP and mild or moderate bleeding may be managed expectantly (“watch and wait”). The International Consensus Report recommended that, when treatment is required in children with newly diagnosed ITP, first-line treatment should include corticosteroids together with IVIg or anti-D in patients with moderate or severe bleeding [7]. It was noted that corticosteroids should be used for the shortest time possible in children and should be tapered and stopped by 3 weeks of therapy if a response is seen, to avoid the side effects associated with prolonged treatment. For emergency treatment in children at any stage of ITP, combination therapy was recommended (e.g., high doses of corticosteroids, IVIg, and platelet transfusions); in these patients, TPO-RAs may also be considered, as they may aid the acute response in patients and prevent a decrease in platelet count if initial response to therapy is lost.

In children with newly diagnosed ITP who have no or minor bleeding, the ASH guideline panel suggests observation rather than corticosteroids [17]. The guidelines recommended corticosteroids for the standard first-line treatment of children with newly diagnosed ITP who have non-life-threatening mucosal bleeding and/or diminished HRQoL. Long-term corticosteroid therapy was discouraged, and a short course of ≤ 7 days was recommended in children. The guidelines suggested TPO-RAs as the primary second-line treatment of choice in children at any stage of ITP who do not respond to first-line treatment.

The guidelines from the joint working group of hematology societies in Germany, Austria, and Switzerland state that treatment is usually not recommended for newly diagnosed ITP in children and adolescents with no or only mild bleeding [6]. In this scenario, a watch and wait strategy is appropriate; low platelet counts alone are not an indication to initiate treatment in children or adolescents with newly diagnosed ITP, and several additional factors should be considered, including clinical bleeding tendency, side effects, consequences for school, access to care, patient preference, and importantly, the risk of injury during leisure activities. If pharmacological intervention is deemed appropriate, corticosteroids are the recommended first line of treatment. IVIg should be used for heavy bleeding (with additional platelet concentrates for very heavy bleeding), as these achieve a faster rise in platelet counts than corticosteroids alone. It was recommended that TPO-RAs and rituximab should be considered in cases of life-threatening bleeding if IVIg and corticosteroids do not achieve hemostasis. TPO-RAs were also recommended as the second-line therapy of choice, even if the disease duration was not yet a year. While this recommendation was not specific to children, the authors noted that TPO-RAs were equally effective in old and young patients and that they were better tolerated than corticosteroids or other ITP treatments such as IVIg and anti-D.

### Clinical trials

No randomized clinical trial data for romiplostim in children with newly diagnosed ITP were identified.

### Real-world studies and case reports

Several real-world studies and case reports have assessed romiplostim in children with newly diagnosed ITP [29–34] (Table 1). The study with the largest cohort of children with newly diagnosed ITP who received romiplostim was a retrospective multicenter chart review, performed at 12 sites in the Pediatric Immune Thrombocytopenia Consortium of North America (ICON) [29]. The study included a total of 79 children with newly diagnosed, persistent, or chronic ITP; of these, 51 received romiplostim with 13/51 having newly diagnosed ITP. Overall, 44/51 (86%) of children achieved a platelet count ≥ 50 × 10^9^/l at least once in the absence of rescue therapy during the first 3 months with romiplostim. Additionally, 37/51 (73%) of romiplostim-treated patients achieved a platelet count ≥ 20 × 10^9^/l above baseline for 2 consecutive weeks without requiring new or increased concomitant ITP treatment; the average time to a consecutive response in these patients was 6.4 weeks. While only combined data for the three phases of ITP was presented, the authors stated that there was no difference in duration of ITP between responders and non-responders. Romiplostim was also well tolerated, and the only significant adverse event noted was the development of neutralizing antibodies in one patient.

**Table 1:** Use of romiplostim in real-world studies including patients with newly diagnosed or persistent ITP [29–34]

Reference	Country	Study design	Patients treated with romiplostim (newly diagnosed or persistent ITP only)	Results
Neunert et al. (2016) [29]	USA	Retrospective multicenter case review (N = 79)	Newly diagnosed, n = 13 Persistent, n = 10	• Overall (incl. chronic ITP), 86% of children achieved a platelet count ≥ 50 × 10^9^/l with romiplostim at least once • 73% achieved a platelet count ≥ 20 × 10^9^/l above baseline for 2 consecutive weeks without requiring new or increased concomitant ITP treatment
Ramaswamy et al. (2014) [30]	USA	Retrospective observational study (N = 33)	Newly diagnosed, n = 2 Persistent, n = 5	• Both children with newly diagnosed ITP and 4/5 with persistent ITP showed a platelet response ≥ 50 × 10^9^/l • Newly diagnosed: 1/2 achieved a CR; 1/2 achieved a PR • Persistent: 3/5 achieved a CR; 1/5 achieved a PR
Suntsova et al. (2020) [31]	Russia	Retrospective observational study (N = 6)	Newly diagnosed, n = 6	• CR was achieved in 5/6 patients 4–8 weeks after initiating therapy • Three children remained in remission for 1–3 years after the discontinuation of romiplostim
Grace et al. (2019) [32]	USA	Prospective observational study (N = 120)	Newly diagnosed, n = 6 Persistent, n = 9	• Overall (incl. chronic ITP) at month 6, 71% of patients achieved a CR and 15% achieved a PR
Suntsova et al. (2017) [33]	Russia	Prospective observational study (N = 20)	Newly diagnosed, n = 1 Persistent, n = 3	• Newly diagnosed: platelet count increased from 3 × 10^9^/l to 16 × 10^9^/l after treatment with romiplostim and eltrombopag; the patient was deemed a non-responder to both treatments • Persistent: 2/3 were considered responders to romiplostim
Escudero Vilaplana et al. (2012) [34]	Spain	Retrospective case report (N = 3)	Newly diagnosed, n = 1	• The time for CR was 14 days, and response was maintained for 6 months • Median platelet count was 215 × 10^9^/l over the 6 months

*CR*, complete response; *ITP*, immune thrombocytopenia; *PR*, partial response

Two other retrospective analyses assessing romiplostim have been conducted that included children with newly diagnosed ITP [30, 31]. Ramaswamy et al. (2014) [30] included a total of 33 children treated with TPO-RAs in the USA; of these, two male patients had newly diagnosed ITP (ITP duration 2 and 3 months, respectively) and received treatment with romiplostim. Baseline platelet counts in the individuals with newly diagnosed ITP were 3 × 10^9^/l and 11 × 10^9^/l, prior to treatment. Both children showed a platelet response ≥ 50 × 10^9^/l with romiplostim, with one achieving a complete response and the other a partial response. No serious drug-related adverse events occurred with romiplostim in this study. Suntsova et al. (2020) [31] included six children (n = 5 male, n = 1 female; ages 3 months to 7 years) with newly diagnosed ITP who received romiplostim. Baseline platelet counts ranged from 1 × 10^9^/l to 7 × 10^9^/l, and all patients had previously received corticosteroids and IVIg; five patients had previously received platelet concentrates and two had received red blood cell suspensions. Five of six children achieved a durable response (platelet count > 100 × 10^9^/l) 4–8 weeks after starting therapy, and three children remained in lasting remission for 1–3 years after discontinuation of romiplostim. No adverse events associated with romiplostim use were reported.

Two prospective observational studies with romiplostim have been conducted that included children with newly diagnosed ITP. Grace et al. (2019) [32] included 120 children with ITP requiring second-line treatments, of whom 31 received romiplostim, with six of these having newly diagnosed ITP. Complete and partial responses were found in 71% and 15% of all patients receiving romiplostim at 6 months, respectively. Suntsova et al. (2017) [33] included 20 children, of whom one had newly diagnosed ITP (female; aged 5 years; previously treated with corticosteroids and IVIg) and received eltrombopag followed by romiplostim. The platelet count increased from 3 × 10^9^/l to 16 × 10^9^/l, but the patient was deemed a non-responder to either therapy.

Finally, a case report by Escudero Vilaplana et al. (2012) [34] evaluated romiplostim treatment in three children with ITP. One of these children (male, aged 13 years; on immunosuppressive therapy with tacrolimus and antiplatelet therapy with acetylsalicylic acid; platelet count, 2 × 10^9^/l) had newly diagnosed ITP and showed an initial response to corticosteroids but relapsed and was readmitted a month later. Romiplostim treatment was started 2.2 months after diagnosis and resulted in a complete response for 14 days. The response was maintained for 6 months, during which time the median platelet count was 215 × 10^9^/l.

## Romiplostim for children with persistent ITP

### Guidelines

The recently updated major regional and international guidelines support TPO-RAs for the treatment of children with persistent ITP [6, 7, 17]. The International Consensus Report suggested TPO-RAs as the preferred treatment in children with persistent/chronic ITP in whom alleviating thrombocytopenia is likely to provide a clear clinical benefit [7].

The updated ASH guidelines—as well those from the joint working group of European hematology societies in Germany, Austria, and Switzerland—also recommended TPO-RAs as second-line therapy in all patients that require intervention and who have not responded to first-line treatment [6, 17]. These include patients whose disease duration is < 1 year, although the recommendations were not specific for children with persistent ITP [6, 17].

### Clinical trials

Five clinical trials, including two randomized and three long-term open-label studies, have investigated romiplostim in children (< 18 years of age) who had ITP for ≥ 6 months and therefore included patients with persistent or chronic ITP according to the new definition of the three phases of ITP [21, 22, 25, 27, 28]. While the results from each study were not presented separately for children with persistent versus chronic ITP, the studies demonstrated the efficacy of romiplostim in achieving and maintaining platelet responses in the overall population. Additionally, long-term romiplostim treatment was well tolerated, with a low number of treatment-related serious adverse events reported [21, 22, 25, 27, 28].

A recent study analyzed an integrated database from these five clinical trials, with the results presented separately for children with persistent versus chronic ITP [35, 36] (Table 2). In total, 282 patients received any romiplostim and 24 any placebo, with each group including 20 patients who initially received placebo and then switched to romiplostim. At baseline in the romiplostim group, 69/282 (25%) had persistent ITP, and 213/282 (76%) had chronic ITP. Overall, 89% of children in the romiplostim group had a platelet response (≥ 50 × 10^9^/l), and the results were similar in those with persistent versus chronic ITP (platelet responses of 88% and 90%, respectively). Furthermore, 19/282 (7%) patients (persistent ITP, 10%; chronic ITP, 6%) had a treatment-free response defined as a maintenance of platelet counts ≥ 50 × 10^9^/l for ≥ 6 months while withholding all ITP therapies. Additionally, the study found that romiplostim was well tolerated and no immunogenicity or bone marrow issues were identified. Treatment-related serious adverse events occurred in 2.5% of patients receiving romiplostim (7/282). The frequency of serious adverse events (regardless of causality to treatment) was numerically slightly lower in the persistent versus chronic ITP group (17% vs 27%, respectively). The most frequently reported serious adverse events in the overall romiplostim group (epistaxis [5.7%], decreased platelet count [2.5%], and thrombocytopenia [2.5%]) were not unexpected for children with ITP regardless of treatment [36]. Overall, this large, integrated analysis of five clinical trials supports the established safety profile of romiplostim in children.

**Table 2:** Efficacy and safety of romiplostim in children with persistent or chronic ITP: integrated analysis of five clinical trials [35, 36]

	Persistent ITP	Chronic ITP
	Any romiplostim (n = 69)	Any romiplostim (n = 213)
Efficacy		
Platelet response (≥ 50 × 10^9^/l), %	88	90
Median time to platelet response, weeks	5	6
Median proportion of time/month with platelet response, %	81	74
Treatment-free response, %	10	6
Median duration of treatment-free response, months	12	11
Safety		
Median duration of treatment, weeks	65	66
Serious adverse events, %	17	27

*ITP*, immune thrombocytopenia

### Real-world studies and case reports

A number of real-world studies have included children with persistent ITP in addition to those with chronic disease (see Table 1). The retrospective multicenter case review by Neunert et al. (2016) [29] included 10 children with persistent ITP out of the total of 51 patients who received romiplostim. As mentioned in the newly diagnosed ITP section, the combined results for all children were reported as 86% achieving a platelet count ≥ 50 × 10^9^/l with romiplostim at least once, with no difference in the duration of ITP between responders and non-responders. The retrospective study by Ramaswamy et al. (2014) [30] included five children with persistent ITP (n = 4 female, n = 1 male; baseline platelet count range, 2–10 × 10^9^/l) who received romiplostim; of these, four had a platelet response ≥ 50 × 10^9^/l, with three achieving a complete response and one a partial response. Similarly, Suntsova et al. (2017) [33] included three children with persistent ITP (platelet count range, 6–12 × 10^9^/l before treatment) who received romiplostim; two of these were considered responders to romiplostim. Finally, Grace et al. (2019) [32] included nine patients with persistent ITP; the results were presented for the overall romiplostim-treated population (n = 31), and as mentioned previously, 86% of patients achieved a complete or partial response at 6 months.

## Reflection on the role of romiplostim for children with newly diagnosed or persistent ITP and future perspectives

Randomized clinical trial data for romiplostim are lacking particularly for children with newly diagnosed ITP, and there a number of important research topics to be considered (Table 3). These include efficacy and safety of TPO-RAs in this patient population, as well as the optimal timing of treatment with respect to TPO-RAs versus other approaches. While limited pharmacoeconomic data are available (see Cost-effectiveness section), further studies are required to understand the cost–benefit of early TPO-RA use versus other approaches. Additionally, other important questions include whether there is distinct subgroup of children who will benefit from early TPO-RA use, if there are markers to identify this subgroup, and if there is a clinical advantage to the early use of TPO-RAs versus corticosteroids and “watch and wait.”

**Table 3:** Key remaining questions on the role of romiplostim for children with newly diagnosed or persistent ITP

Area/topic	Key questions
Biological/clinical questions	• Is there a subgroup of children who benefit most from the early use of TPO-RAs?
	• Is the early use of TPO-RAs associated with a changed incidence of chronic ITP or altered course of ITP?
Questions to be answered in interventional trials	• What is the efficacy of TPO-RAs in children with newly diagnosed ITP?
	• In children with newly diagnosed ITP, should a first attempt with corticosteroids be undertaken before using TPO-RAs in those who do not respond or who respond inadequately?
	• Can corticosteroids be omitted if TPO-RAs are used in children with newly diagnosed ITP? Could this strategy lead to a reduction in the overuse of corticosteroids? Conversely, could it lead to an overuse of TPO-RAs?
	• What are the relative benefits of TPO-RAs versus “watch and wait”?
	• How should children who do not respond to TPO-RAs be managed?
	• What is the safety profile associated with the early use of TPO-RAs in children?
Pharmacoeconomic questions	• Is there a pharmacoeconomic benefit or otherwise to the early use of TPO-RAs in children?

*ITP*, immune thrombocytopenia; *TPO-RAs*, thrombopoietin receptor agonists

Despite the lack of randomized clinical trial data and remaining questions, the collective body of evidence, which includes real-world studies and case reports, supports the early use of romiplostim in children who have received prior corticosteroid or IVIg treatment. Overall, the available efficacy data indicate that a high proportion of children with newly diagnosed and persistent ITP achieve platelet responses, with responses appearing similar to those reported for chronic ITP [29, 35, 36]. Furthermore, the available safety and tolerability data from studies that included children with newly diagnosed or persistent ITP identified no major concerns, thus supporting the well-characterized safety profile in children with chronic ITP. However, while the available efficacy and safety evidence support the use of romiplostim earlier in the course of ITP, it is important to note that its use for the treatment of children with newly diagnosed or persistent ITP is currently off-label in Europe (as is its first-line use in these populations) and so may not be reimbursed.

Importantly, there is some evidence that patients with newly diagnosed or persistent ITP develop treatment-free durable platelet responses or lasting remission after treatment with romiplostim [31, 35, 36]. While spontaneous remission is common in children at this stage of disease, it has been speculated that the various biological actions of romiplostim may positively affect remission rates [18, 37]. It has been postulated that romiplostim could restore immune tolerance by increasing exposure to platelet antigens, thereby reducing platelet antibodies [37, 38]. Additionally, some information suggests that increased presence of platelets following romiplostim and other TPO-RA treatments may lead to an increase in the serum levels of TGF-β, which could improve regulatory T cell function and immune tolerance [18, 39]. The molecular mechanisms by which romiplostim mediates its effects on the immune system are not fully understood, but its Fc fragment might play a role. Fc fragments of other immunogenic drugs have been reported to confer tolerogenic effects, although it should be noted that the importance of the Fc fragment of romiplostim has not yet been fully investigated [18]. Overall, further studies are required to investigate the extent to which romiplostim can lead to long-term treatment-free responses in children with newly diagnosed or persistent ITP and the mechanisms behind this effect.

### First-line treatment of children with newly diagnosed or persistent ITP

Several first-line management options have been used for children with newly diagnosed or persistent ITP. Observation (“watch and wait”) is recommended in children with newly diagnosed ITP with mild-to-moderate bleeding if it has a minimal impact on HRQoL [7, 17]; however, observation is less validated in children with persistent ITP, as it is based on the expectation of spontaneous future improvement [7]. When treatment is required for children with newly diagnosed ITP, corticosteroids have been used as the standard first-line therapy due to their effectiveness in increasing platelet counts in the short term [6, 7, 17]. However, long-term corticosteroids have been associated with a range of serious adverse effects including hypertension, hyperglycemia, gastritis, and mood changes [40]. Furthermore, corticosteroids can cause a range of less serious side effects that could negatively affect HRQoL [41], which may be a particular concern in children. As a result, corticosteroids should be used for as short a time as possible in children, with the ASH guidelines recommending against courses longer than 7 days [17], the International Consensus Report recommending stopping by 3 weeks including taper [7], and the joint working group of European hematology societies in Germany, Austria, and Switzerland guidelines recommending no longer than 2 weeks [6]. Other first-line treatments for children with severe bleeding include IVIg and anti-D, which have the benefit of rapidly increasing platelet counts but frequently demonstrate only transient responses [40]. Furthermore, these treatments are associated with serious, albeit transient, side effects, including infusion reactions, headaches, aseptic meningitis, and hemolysis [40].

Overall, first-line treatment for patients with diagnosed or persistent ITP should be personalized and prevent severe bleeding episodes (by maintaining target platelet levels > 20–30 × 10^9^/l) while having minimal toxicity and improving HRQoL [7]. Current clinical management strategies of ITP are associated with a substantial healthcare burden and an effect on the HRQoL of children [13, 42], and there is an overreliance on corticosteroids in clinical practice [7]. It is possible that TPO-RAs such as romiplostim may help children avoid side effects associated with long-term corticosteroid use and treatment with IVIg and anti-D during acute bleeding episodes. Furthermore, the earlier use of TPO-RAs may represent a more efficient use of healthcare resources than the current standard practice [42], although no specific studies have examined this in children.

An aim of first-line therapy for patients with newly diagnosed or persistent ITP should be the prevention of long-term chronic disease where possible; however, this is not achieved in most patients using corticosteroids or IVIg. The high relapse rate of patients on corticosteroids suggests that these do not shorten the disease course, and evidence from a randomized clinical trial indicates that the use of IVIg in children with newly diagnosed ITP might not decrease the rate of developing chronic disease [43, 44]. The extent to which TPO-RAs lead to long-term treatment-free durable platelet responses or lasting remission in children with newly diagnosed or persistent ITP thus warrants further investigation. However, before clinicians consider the use of TPO-RAs in the first-line setting, further studies are required to directly compare the efficacy and safety of TPO-RAs versus the current first-line standard of care in children with newly diagnosed ITP.

### Second-line treatment of children with newly diagnosed or persistent ITP

As previously mentioned, the collective body of evidence supports the use of romiplostim for second-line treatment in children with newly diagnosed or persistent ITP. Other than romiplostim, the only other TPO-RA currently approved for use in children in Europe is eltrombopag, which is indicated for the treatment of patients ≥ 1 year of age for ITP lasting ≥ 6 months from diagnosis and who are refractory to other treatments [45]. Eltrombopag is an oral drug taken daily, compared with romiplostim which is administered by weekly subcutaneous injection [19, 45]. While oral dosing may be more convenient for patients, eltrombopag absorption has been shown to be severely impacted by some dietary components [46, 47]. As a result, it should be taken ≥ 2 h before, or 4 h after, calcium-containing food products [45]. Overall, patients can have varying responses to different TPO-RAs; further studies including comparative clinical trials between romiplostim and eltrombopag are warranted to help guide second-line treatment decisions.

The most recent TPO-RA in clinical use, avatrombopag, is approved in Europe for the treatment of primary chronic ITP in adult patients who are refractory to other treatments (e.g., corticosteroids and immunoglobulins) [48]. It is also approved in the USA for the treatment of adult patients with chronic ITP who have had an insufficient response to a previous treatment [49].

Other subsequent management options for children with ITP include rituximab, which is currently not approved for the treatment of ITP and splenectomy. The ASH guidelines suggest the use of TPO-RAs rather than rituximab for second-line therapy in children [17], while the International Consensus Report recommends consideration of rituximab in children with persistent/chronic ITP only in those that first fail on TPO-RAs [7]. Additionally, TPO-RAs avoid the immunosuppression risks associated with rituximab [40]. Finally, splenectomy is now very rarely indicated in children with ITP and should only be considered in those that fail all available therapies, given the ongoing lifelong risks following the procedure [7, 17].

Clinical decision-making for the selection of second-line treatments has been investigated by the ICON1 study, which included 120 children with ITP; of these, 16% and 31% had newly diagnosed and persistent ITP, respectively [50]. Clinicians indicated expected efficacy as a reason for choosing romiplostim versus other second-line agents, including eltrombopag, while parental or patient preference was also important for choosing romiplostim. The perceived side-effect profiles were another key reason for choosing romiplostim and eltrombopag rather than rituximab and oral immunosuppressants. A further analysis of the ICON1 study cohort evaluated the effect of the second-line agents on fatigue [15]. Overall, fatigue significantly improved in children and adolescents while taking the second-line treatments. When individual treatments were analyzed separately, rituximab significantly reduced fatigue, while there was a trend for a reduction with romiplostim and eltrombopag.

Recent data suggest that the TPO-RAs are increasingly being used for the second-line treatment of ITP in children. A retrospective review of second-line treatments for persistent or chronic ITP from the UK pediatric ITP registry evaluated the changing pattern of treatment between 2006 and 2019 [51]. Out of the 1915 children on the registry during this period, 212 were eligible for second-line therapy, and 23% of these received treatment. The use of TPO-RAs increased from 23% (2006–2011) to 48% (2015–2019) and was the most frequently used second-line therapy during the latter time period compared with rituximab (24%) and splenectomy (9.5%).

Collectively, the available data on clinical decision-making and changing treatment patterns support the efficacy and safety results from clinical studies, as well as guideline recommendations that TPO-RAs may be a preferred choice for children with newly diagnosed or persistent ITP that fail on corticosteroid therapy. The selection of a particular TPO-RA for an individual child should be made together with the patient/parent, based on preference (i.e., for an oral or once-weekly injected product), totality of evidence, cost, and adverse events.

### Cost-effectiveness considerations

To our knowledge, no health economic analyses have been conducted to determine the cost-effectiveness of romiplostim for treating children and adolescents with newly diagnosed or persistent ITP. In adult patients with chronic ITP, romiplostim has been shown to be cost-effective [52–54]. For example, from an Irish healthcare perspective, romiplostim was estimated to provide cost savings of €22,673 and gains of 1.17 quality-adjusted life years compared with standard of care in adults with chronic ITP [54]. Savings were driven by higher response rates associated with romiplostim, which led to a reduction in bleeding events and less use of rescue therapies [54]. Another adult study in chronic ITP also suggested that romiplostim had lower costs per response than “watch and rescue” [55]. However, in pediatric chronic ITP, a cost–consequence analysis suggested that the cost per patient could be higher for romiplostim than for watch and rescue [56]. It should be noted that there are a number of limitations with cost-effective analyses; not least the generalizability of the results and that the setting in which romiplostim is administered to patients via subcutaneous injection may vary depending on the country. Overall, further analyses are warranted, particularly in the context of evaluating the cost/benefits associated with avoiding long courses of corticosteroids and assessing the potential long-term benefits of early treatment with TPO-RAs.

### Potential impact of COVID-19 on the treatment landscape of children with ITP

The treatment of ITP should be considered together with the risks of COVID-19. Several societies have issued guidance for COVID-19 and patients with ITP [57–61]; however, specific guidance for children is limited. The available guidance suggests caution when using immunosuppressive agents, such as corticosteroids and rituximab, because of the potential risk of infection. This may be more applicable in at-risk adults, as COVID-19 is usually a mild self-limiting illness in children, even in those who are immunocompromised [62]. While immunosuppression may increase the risk for severe COVID-19 illness in children [63], clinical data demonstrating this are lacking [64]. Nevertheless, the National Institute for Health and Care Excellence (NICE) guidance states that alternatives to immunosuppressive agents with a lower risk for COVID-19 should be considered for children [62]. Additionally, children taking corticosteroids may have an atypical presentation of COVID-19 [62], which has the potential to result in a delayed diagnosis. Overall, the perceived risks associated with immunosuppression may increase the usage of alternative treatments for children with ITP; however, further studies are required to investigate whether short-term and long-term corticosteroid use increase the risks associated with COVID-19 in children.

Another area for future research is whether COVID-19 has an impact on ITP in children and if treatment needs to be modified in these patients accordingly. There is evidence to suggest that COVID-19 may affect platelet counts and increase the risk of thrombotic complications in adults [65–67], but there are currently no available data in children.

## Conclusions

In Europe, romiplostim is currently indicated for the treatment of children with chronic ITP who are refractory to other treatments. However, the available evidence presented in this narrative review suggests that romiplostim is also efficacious and well tolerated in children with ITP < 1 year from diagnosis. Randomized clinical studies originally investigating romiplostim included children with persistent ITP (following the updated definition of the three phases of ITP), and therefore, the evidence is particularly robust in these patients versus those with newly diagnosed ITP. As a result of data from these randomized clinical studies, romiplostim (as well as other TPO-RAs) is generally recommended in guidelines for children with ITP who fail on first-line therapy, including in those with ITP < 1 year from diagnosis. Earlier treatment with TPO-RAs may help children to avoid the side effects associated with prolonged corticosteroid use. While narrative reviews are inherently susceptible to biases, the results presented herein provide the foundation for future systematic reviews and meta-analyses into the efficacy and safety of early TPO-RA use in children. Further studies are warranted to investigate the optimal sequence and timing of management options for children with newly diagnosed and persistent ITP.

## Data Availability

Not applicable.
